# Early Endogenous Activation of CB1 and CB2 Receptors after Spinal Cord Injury Is a Protective Response Involved in Spontaneous Recovery

**DOI:** 10.1371/journal.pone.0049057

**Published:** 2012-11-13

**Authors:** Angel Arevalo-Martin, Daniel Garcia-Ovejero, Yolanda Sierra-Palomares, Beatriz Paniagua-Torija, Ines Gonzalez-Gil, Silvia Ortega-Gutierrez, Eduardo Molina-Holgado

**Affiliations:** 1 Laboratorio de Neuroinflamacion, Hospital Nacional de Paraplejicos, SESCAM, Toledo, Spain; 2 Departamento de Química Orgánica I, Facultad de Ciencias Químicas, Universidad Complutense de Madrid, Madrid, Spain; Hertie Institute for Clinical Brain Research, University of Tuebingen, Germany

## Abstract

Spinal cord injury (SCI) induces a cascade of processes that may further expand the damage (secondary injury) or, alternatively, may be part of a safeguard response. Here we show that after a moderate-severe contusive SCI in rats there is a significant and very early increase in the spinal cord content of the endocannabinoids 2-arachidonoylglycerol (2-AG) and arachidonoyl ethanolamide (anandamide, AEA). Since 2-AG and AEA act through CB1 and CB2 cannabinoid receptors, we administered at 20 minutes after lesion a single injection of their respective antagonists AM281 and AM630 alone or in combination to block the effects of this early endocannabinoid accumulation. We observed that AM281, AM630 or AM281 plus AM630 administration impairs the spontaneous motor recovery of rats according to the Basso-Beattie-Bresnahan (BBB) locomotor scale. However, blockade of CB1, CB2 or both receptors produced different effects at the histopathological level. Thus, AM630 administration results at 90 days after lesion in increased MHC-II expression by spinal cord microglia/monocytes and reduced number of serotoninergic fibres in lumbar spinal cord (below the lesion). AM281 exerted the same effects but also increased oedema volume estimated by MRI. Co-administration of AM281 and AM630 produced the effects observed with the administration of either AM281 or AM630 and also reduced white matter and myelin preservation and enhanced microgliosis in the epicentre. Overall, our results suggest that the endocannabinoids acting through CB1 and CB2 receptors are part of an early neuroprotective response triggered after SCI that is involved in the spontaneous recovery after an incomplete lesion.

## Introduction

Spinal cord injuries (SCI) result from contusion, compression, stretch or laceration of the spine, being the most frequent contusive/compressive injuries by fractured or dislocated spinal column. However, damage to the cord is not only the result of the initial trauma, but also a consequence of the cascade of cellular and molecular events occurring during the first minutes to days after the injury [1]. This complex secondary injury is a major determinant of final lesion extension and may be the first target for a therapeutic intervention after SCI. In fact, many preclinical studies and most of the clinical trials for SCI are directed to limit the secondary injury in order to prevent neurological function loss and to provide the anatomical substrate for further reparation [2]. For instance, several experimental therapeutic strategies are directed to interfere with all the events related with hypoxia/ischemia and the subsequent ATP depletion, ion pumps malfunction, intracellular calcium accumulation and, finally, excitotoxicity. But not all the events triggered after SCI are involved in augmenting the lesion. On the contrary, some endogenous responses might counteract the detrimental events and fostering them could be useful to reduce secondary injury.

The endocannabinoid system is composed of two types of G protein-coupled receptors (the CB1 and CB2 receptors), the endogenous ligands for these receptors (arachidonoyl ethanolamide or anandamide and 2-arachidonoylglycerol) and the specific enzymatic machinery for their synthesis and degradation [3]. Endocannabinoids are not stored in cells but they are produced on-demand from membrane lipid precursors in response to cell activation. Upon abnormal high spiking activity, this is a protective mechanism against otherwise subsequent excitotoxic damage [4]. In line with this, the endocannabinod system is modulated in response to a variety of neurological insults and its enhancement or the activation of cannabinoid receptors may have therapeutic effects [5]–[10].

We have previously shown that SCI induces a local and transient increase of anandamide levels at 1 day after injury and a delayed increase of 2-AG levels at 7 and 28 days [11]. Also, we have reported that a single injection of 2-AG 30 minutes after lesion protects white matter from secondary damage [12]. In the present study we show that i) after SCI the endocannabinoids 2-AG and anandamide acumulate in the spinal cord earlier than previously described, observing an acute peak of 2-AG levels at 4 hours after injury, and ii) blocking CB1 and/or CB2 receptors impairs the spontaneous functional recovery by augmenting tissue damage.

**Figure 1:**
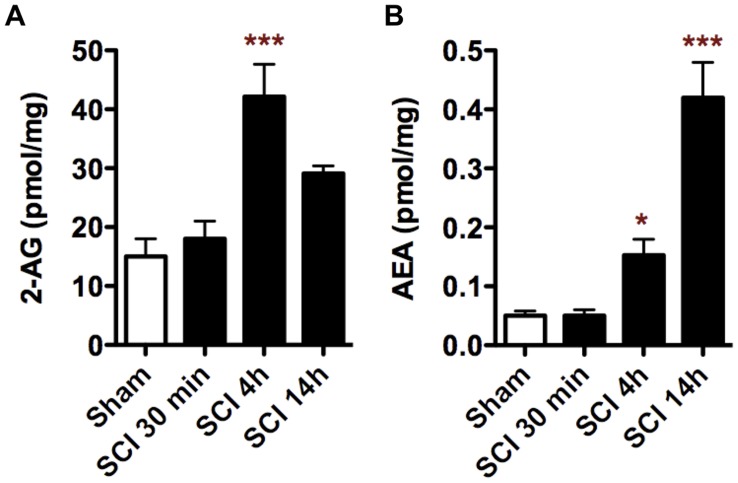
Endocannabinoids are rapidly produced around the epicentre in response to SCI. 2-AG levels are acutely increased at 4 hours after SCI (A), while AEA levels are significantly increased at 4 and, even more, at 14 hours after injury (B). *p<0.05 vs. sham; ***p<0.001 vs. sham.

**Figure 2:**
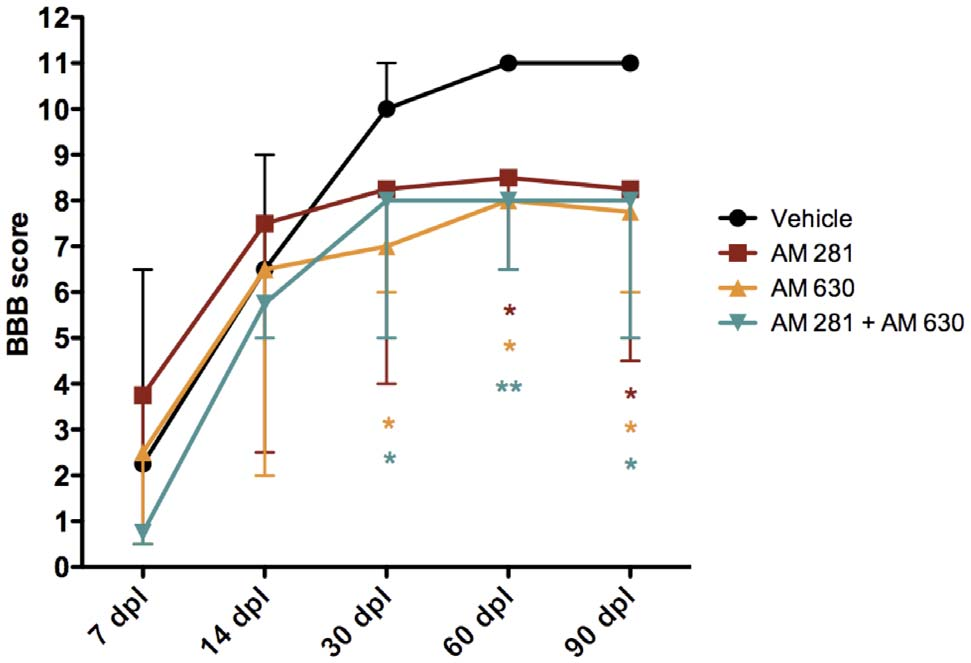
Early pharmacological blockade of CB1 and/or CB2 receptors impairs the spontaneous locomotor improvement after lesion. Evaluation of spontaneous activity using BBB locomotor scale shows that treatment of rats at 20 minutes after lesion with AM281, AM630 or AM281/AM630 impairs recovery. Median values and errors are represented. *p<0.05 vs. vehicle; **p<0.01 vs. vehicle. AM281 n = 6; AM630 n = 5; AM281/AM630 n = 10; vehicle n = 10.

**Figure 3:**
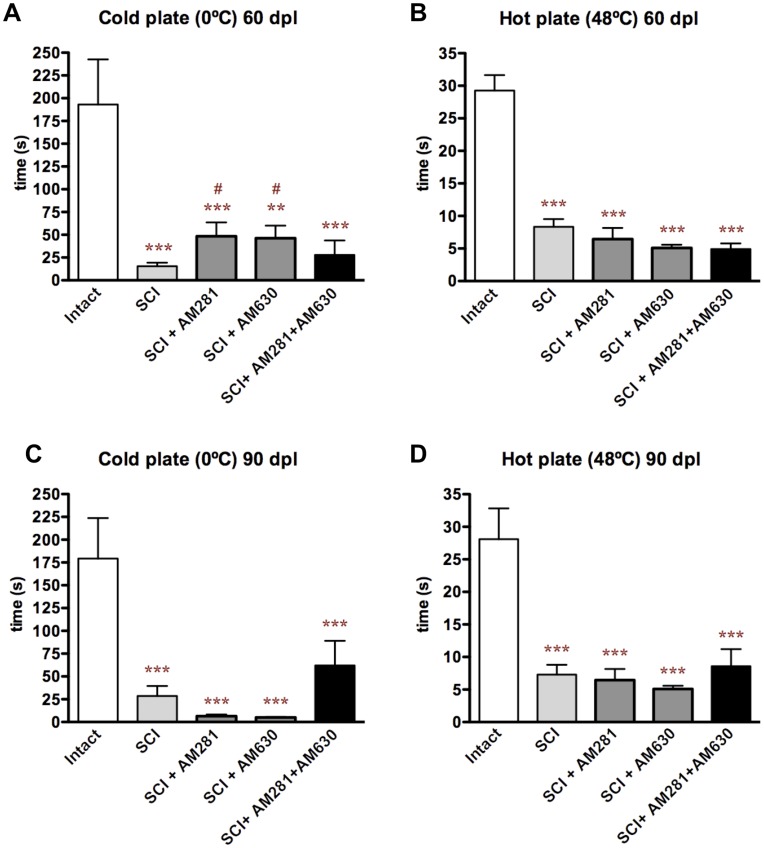
Early blockade of CB1 and/or CB2 receptors does not increase thermal hypersensitivity. Thermal sensitivity to cold and hot plates is represented as the time of withdrawal of hind paw. SCI results in cold and hot hypersensitivity (A-D). We observed a transient decrease of cold hypersensitivity at 60 days after lesion in AM281- or AM630-treated rats (A) that is not further maintained at 90 days after lesion (C). No effect was observed in hot plate test at 60 neither at 90 days after lesion (B, D). ***p<0.001 vs. intact. #p<0.05 vs SCI. Intact n = 4; SCI n = 10; SCI+AM281 n = 6; SCI+AM630 n = 5; SCI+AM281+AM630 n = 10.

**Figure 4:**
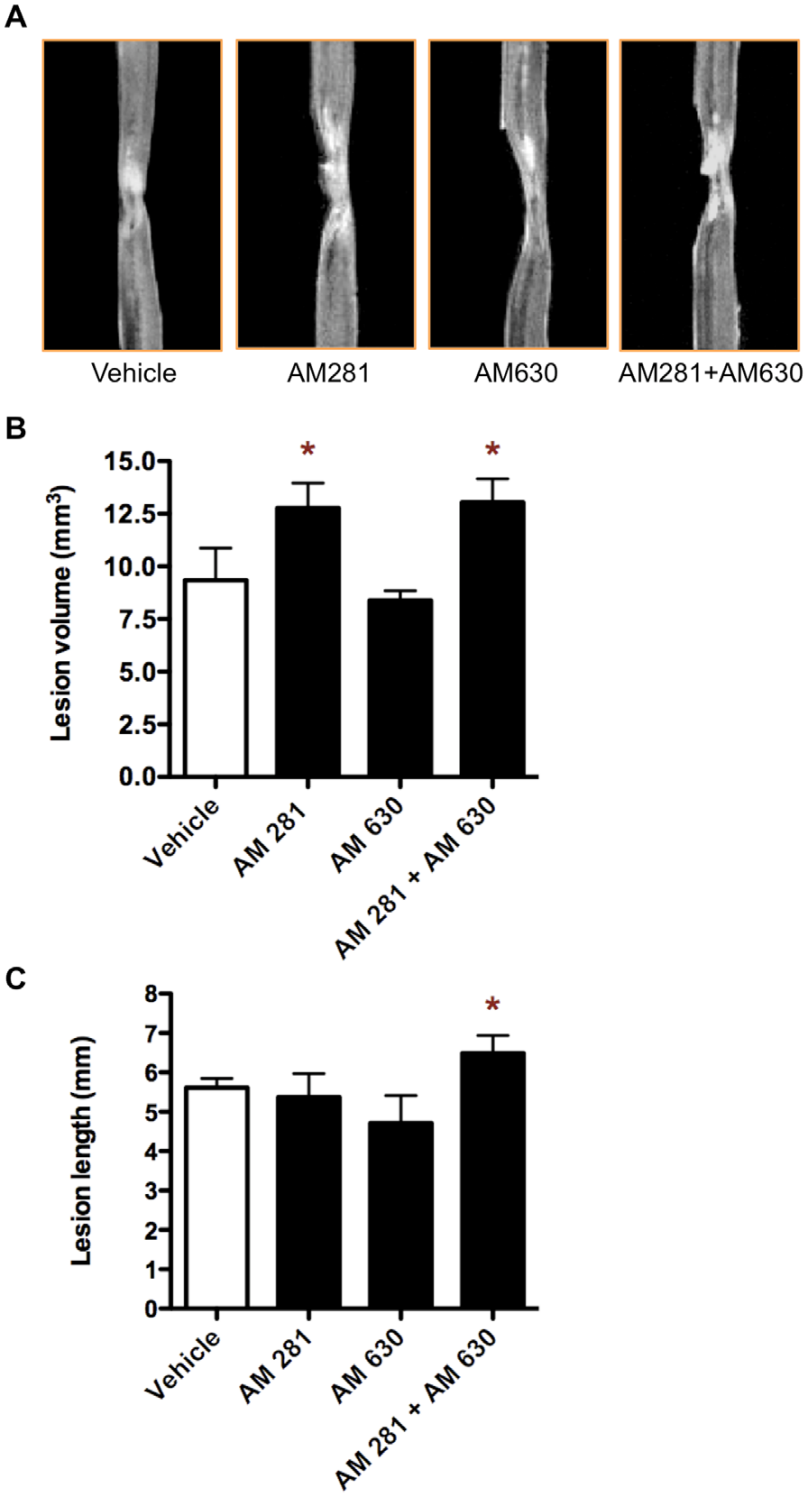
Early blockade of CB1 or CB1 plus CB2 receptors results at long-term in more oedema-related T2 weighted MRI hyperintense signaling. (A) Representative images of 3D-T2 weighted MRI at 90 days after lesion showing the extension of hyperintense signal in vehicle-, AM281-, AM630- and AM281/AM630-treated rats. (B, C) Measurement of the hyperintense signal shows that lesion volume and length are increased in AM281/AM630-treated rats, while lesion volume, but not length, is also greater in AM281-treated rats. *p<0.05 vs. vehicle. Vehicle n = 6; AM281 n = 6; AM630 n = 5; AM281+AM630 n = 5.

## Materials and Methods

### Ethic Statement

Rats were handled in accordance with the guidelines published by Spain and the European Union (RD1201/2005, 86/609/EEC). All experimental procedures were approved by our institutional animal use and care committee, (namely “Comité ético de Bienestar Animal”, approval reference number 40/2008). Postoperative care included analgesia (Buprenorphine) and prophylactic antibiotic treatment (Enrofloxacine), both after injury and on the following day. Hydration was restored during the first week after injury. Manual bladder voiding was employed until the animals recovered self-voiding. The animals were monitored for hydration and eventual infections until the end of the experiment.

### Animals

Young adult male Wistar rats (295–315 g, 12 weeks of age) were obtained from Harlan-Interfauna Ibérica (Barcelona, Spain) and they were maintained in our animal facilities on a 12∶12-hour light:dark cycle, receiving food and water *ad libitum*.

### Spinal Cord Injury (SCI)

We anesthetized rats with an intraperitoneal injection of sodium pentobarbital (45 mg/Kg, Normon Veterinary Division, Madrid, Spain) and Xylacine (10 mg/Kg, Calier, Barcelona, Spain). After confirming the absence of reflexes, we injected atropine (50 µg/Kg, Brown Medical, Barcelona, Spain) and we applied artificial tears to prevent corneal abrasion and infection. We performed a laminectomy of T8 vertebra and we stabilized the vertebral column by clamping spiny processes of T7 and T9 vertebras. Spinal cord contusion/short compression was performed with the “Infinite Horizon™” device, (Precision Systems and Instrumentation, Lexington, KY, USA) applying a force of 200 Kdyn and a compression time of 5 seconds over the exposed cord. Force and displacement curves generated by “Infinite Horizon™” were checked to confirm that injuries were done with similar values and profiles without artefacts indicative of an erroneous/abnormal lesion.

**Figure 5:**
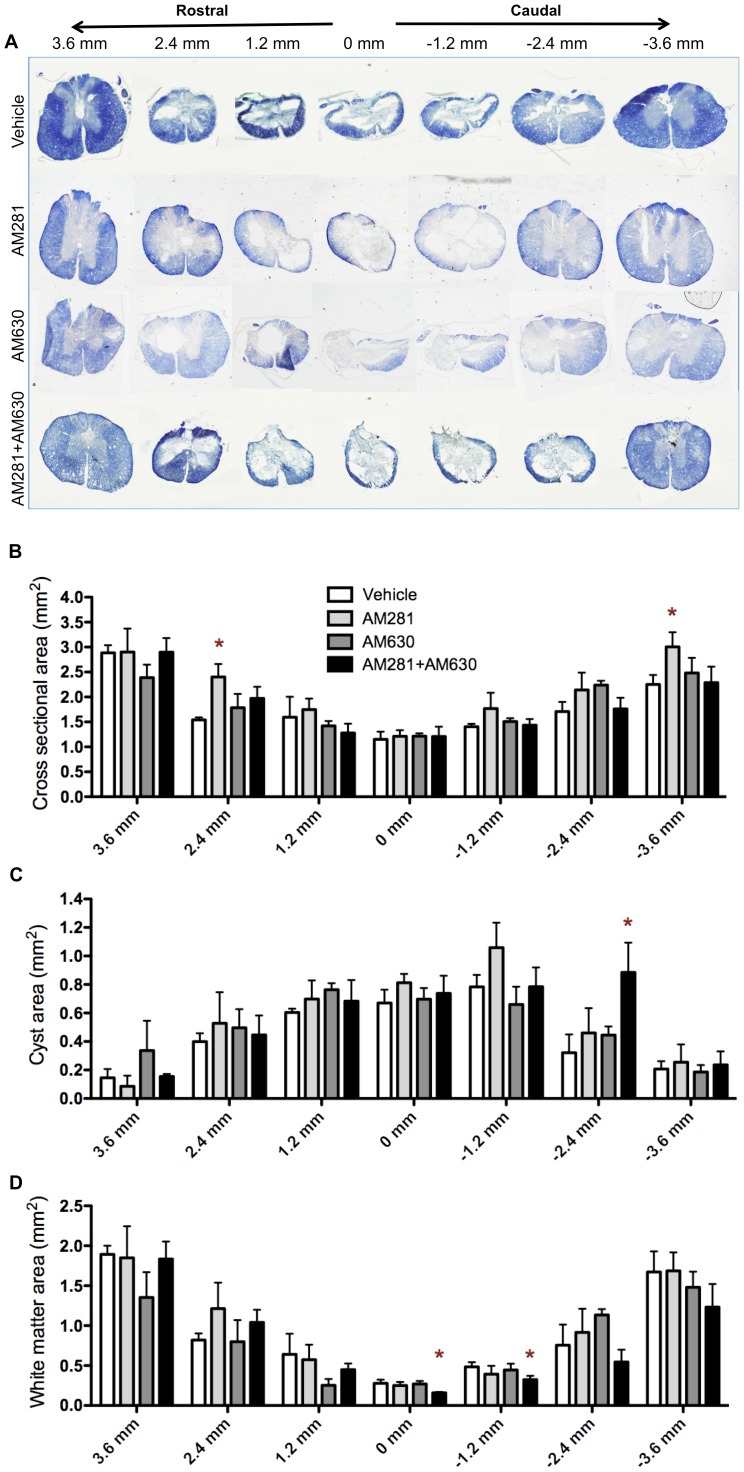
Early blockade of CB1 and CB2 receptors results in more tissue damage 90 days after lesion. (A) Eriochrome cyanine staining of representative spinal cords from vehicle-, AM281-, AM630- and AM281/AM630-treated rat at 90 days after lesion. (B) Quantification of the spinal cord cross sectional area shows no difference between vehicle-, AM630- and AM281/AM630-treated rats. An increase in the cross-sectional area of AM281-treated rats is observed at 2.4 mm rostral and 3.6 mm caudal to the epicentre (C) Evaluation of cyst area reveals that the cyst in AM281/AM630-treated rats remains bigger caudally to the epicentre. (D) In addition, AM281/AM630-treated rats present less white matter preservation at the epicentre and adjacent caudal region. *p<0.05 vs. vehicle. Vehicle n = 6; AM281 n = 6; AM630 n = 5; AM281+AM630 n = 5.

**Figure 6:**
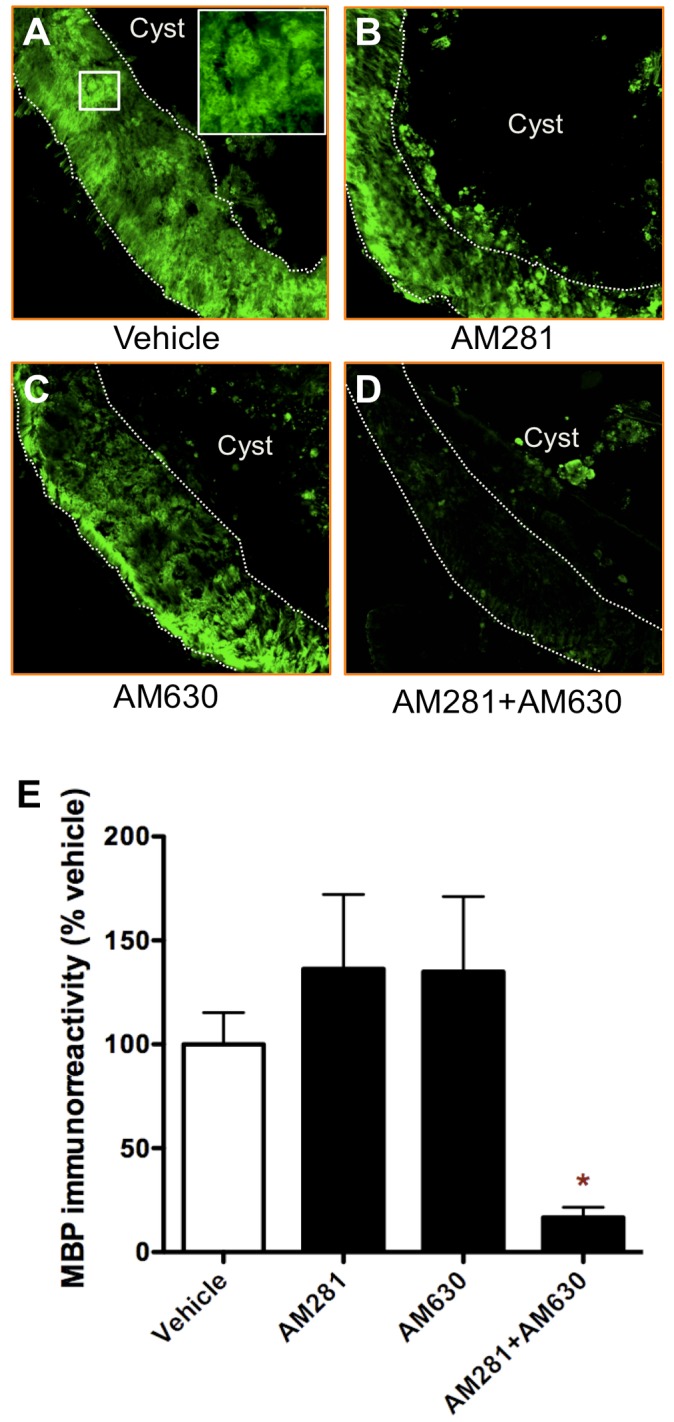
Early blockade of CB1 and CB2 receptors results in less MBP immunoreactivity 90 days after lesion. (A-D) Representative images of MBP immunostaining at lesion epicentre of vehicle-, AM281-, AM630- and 281/AM630- treated rats. (E) Densitometry of MBP immunoreactivity at the epicentre shows that MBP levels are lower in AM281/AM630-treated rats compared to vehicle-treated animals. *p<0.05 vs. vehicle-treated rats. Vehicle n = 10; AM281 n = 6; AM630 n = 5; AM281+AM630 n = 10.

**Figure 7:**
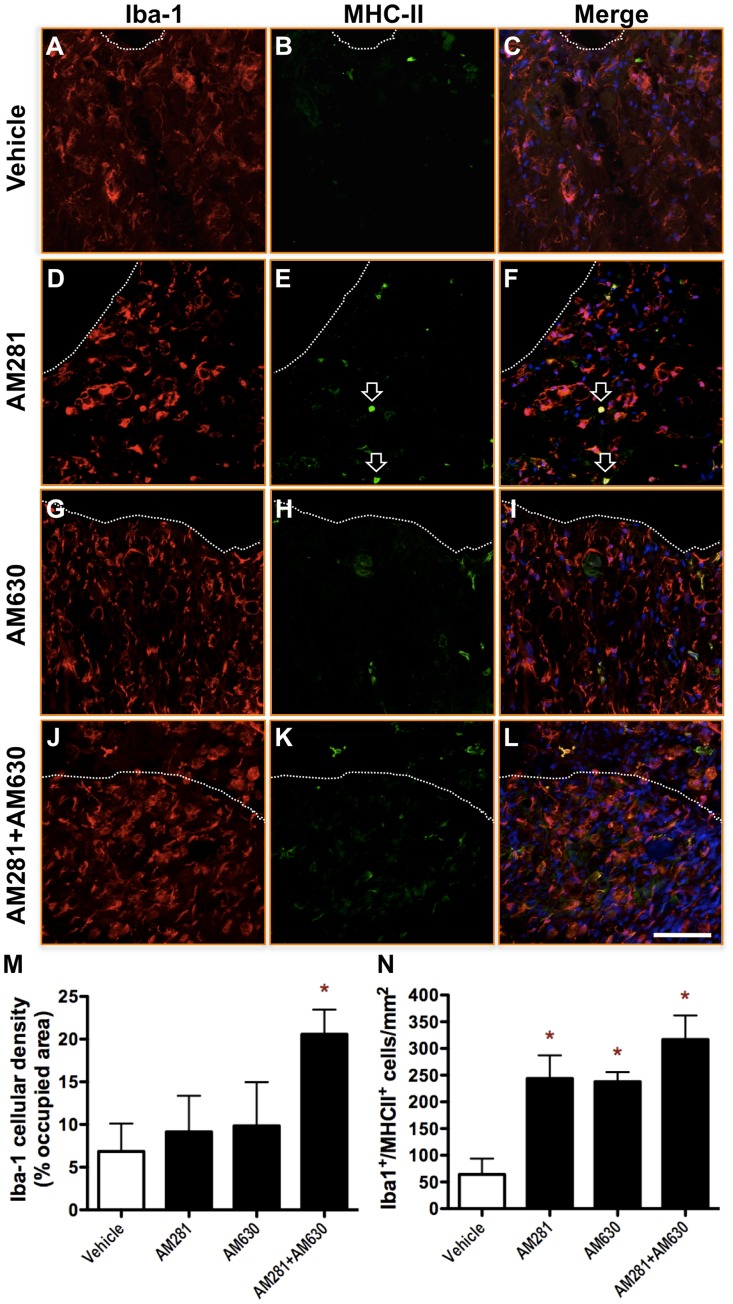
Early blockade of CB1 and CB2 receptors results in increased microgliosis 90 days after lesion. Representative micrographs of microglial reactivity in the spared ventral white matter at 90 days after lesion in vehicle- (A-C), AM281- (D-F), AM630- (G-I) and AM281/AM630-treated rats (J-L). In merge images, nuclear staining is showed in blue. Dashed lines separate spared white matter (below in the images) from cyst (above). AM281/AM630-treated rats exhibited an increased density of Iba-1^+^ cells. (M) Quantification of the area occupied by Iba-1 immunostaining shows a 3-fold increase in AM281/AM630-treated rats when compared to vehicle-treated rats. A small number of Iba-1^+^ cells expressed also MHC-II in the cyst surrounding white matter. (N) However, quantification of double positive cells shows a significant increment in the number of Iba-1^+^/MHC-II^+^ cells in spinal cords of AM281-, AM630- or AM281/AM630-treated rats. Scale bar for all images, 100 µm. *p<0.05 vs. vehicle-treated rats. Vehicle n = 6; AM281 n = 6; AM630 n = 5; AM281+AM630 n = 10.

**Figure 8:**
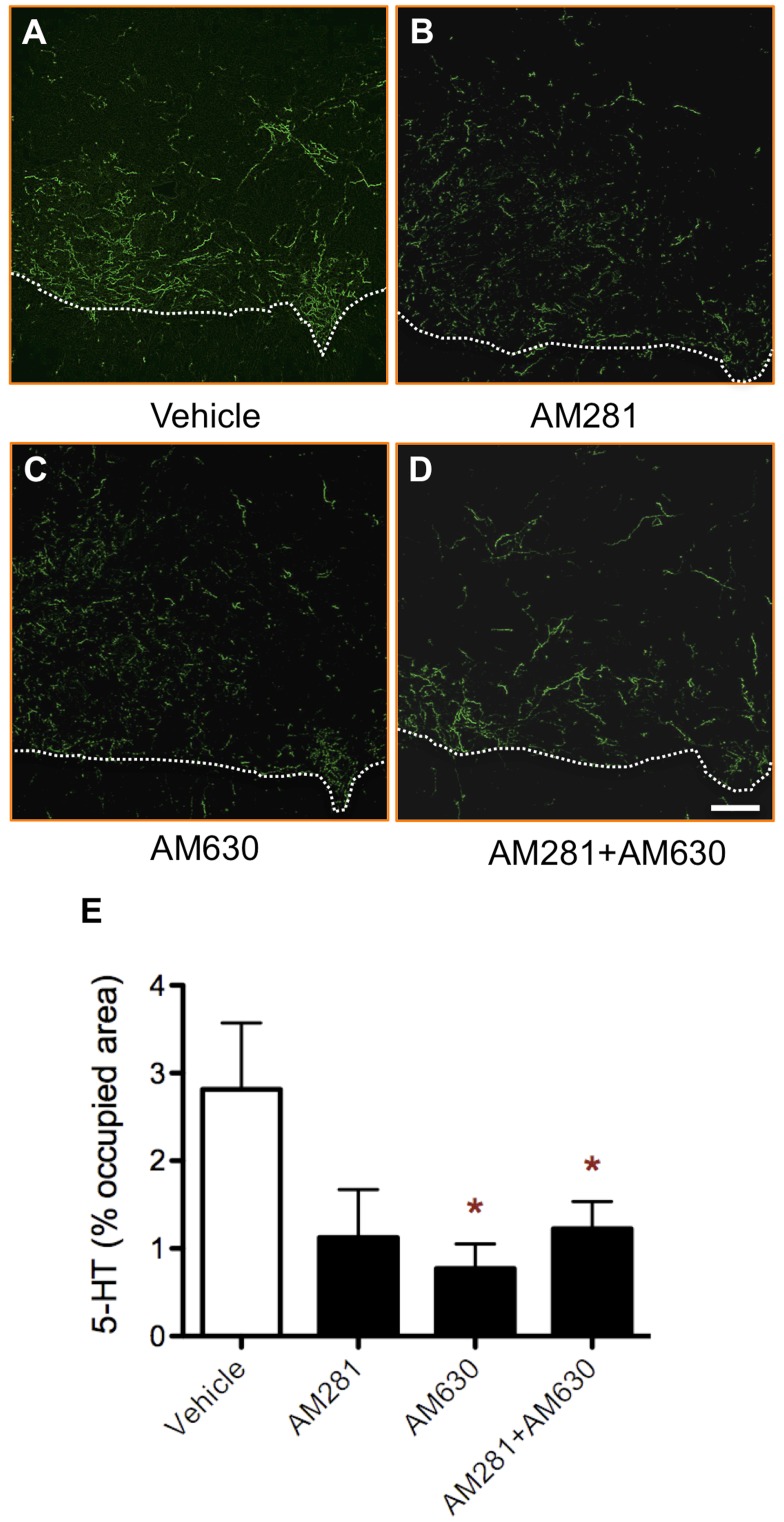
Early blockade of CB1 and/or CB2 receptors reduces the density of serotonergic fibres below the lesion site. Representative serotonin immunostaining of lumbar sections from vehicle- (A), AM281- (B), AM630- (C) and AM281/AM630-treated rats (D). (E) Quantification of the area occupied by serotonin shows a significant decrease in AM630- and AM281/AM630-treated rats and a strong trend to decrease in AM281-treated rats, that is close to statistical significance (p = 0.06). Dashed line separates grey matter (above) from white matter (below). Scale bar for all images, 100 µm. *p<0.05 vs. vehicle-treated rats. Vehicle n = 6; AM281 n = 5; AM 630 n = 5; AM281+AM630 n = 5.

### Quantification of 2-AG and AEA

Tissue samples, stored at −80°C until the moment of analysis, were weighted and homogenized in an ice-cold glass dounce-homogenizer in a mixture 2∶1:1 (v:v:v) of chloroform:methanol:Tris HCl 50 mM (pH  = 7.5). The organic and aqueous layers were separated by centrifugation (4500 *g*, 2 min) and the organic layer transferred to a clean vial and dried under a stream of argon. This fraction was reconstituted in 50 µL acetonitrile and analyzed by high-pressure liquid chromatography coupled to mass spectrometry (LC-MS). LC-MS analysis was performed using an Agilent 1200LC-MSD VL instrument. LC separation was achieved with a Zorbax Eclipse Plus C18 column (5 µm, 4.6 mm×50 mm) together with a guard column (5 µm, 4.6 mm×12.5 mm). The gradient elution mobile phases consisted of A (95∶5 water:methanol) and B (95∶5 methanol:water), with 0.1% ammonium hydroxide and 0.1% formic acid as the solvent modifiers. The gradient (flow rate of 0.5 mL/min) started at 0% B (for 5 min), increased linearly to 100% B over the course of 40 min, and decreased to 0% B for 10 minutes before equilibrating for 5 min with an isocratic gradient of 0% B. MS analysis was performed with an electrospray ionization source. The capillary voltage was set to 3.0 kV, and the fragmentor voltage was set at 70 V. The drying gas temperature was 350°C, the drying gas flow rate was 10 L/min, and the nebulizer pressure was 20 psi. LC-MS measurements were made by selected ion monitoring in positive mode. Fractions were quantified by measuring the area under the peak and normalized using d8-2-AG or d8-AEA (Cayman Chemical) as internal standards. Absolute 2-AG and AEA levels were estimated by comparison with their respective d8-2-AG and d8-AEA standards.

### Treatment of Rats with CB1 and CB2 Receptor Antagonists

Rats were injected intraperitoneally 3 mg/Kg CB1 antagonist/inverse agonist AM281 (Tocris, Bristol, UK; 6 animals), 3 mg/Kg CB2 antagonist/inverse agonist AM630 (Tocris, Bristol, UK; 5 animals), 3 mg/Kg AM281 plus 3 mg/Kg AM630 (10 animals) or the vehicle alone (10 animals) 20 minutes after SCI. Both antagonists were administered in a final volume of 0,5 ml/animal of PBS with 1% BSA. Ten minutes later (30 minutes after injury), animals were placed on a heating pad and 30 minutes later (1 hour after injury), they were subcutaneously injected post-operative analgesics (buprenorphine, 0.05 mg/Kg; Schering Plough, Madrid, Spain) and antibiotics (enrofloxacine, 1 mg/kg; Bayer, Kiel, Germany). After a further 30 minutes (1 hour and 30 minutes after injury), the rats were taken off the heating pads. The temperature of all the animals was monitored each 15 minutes up to 2 hours after the induction of SCI with no differences observed between experimental groups. All AM281- and AM630-treated animals were employed in every study detailed below, while not all vehicle- nor AM281/AM630-treated rats were used in every study; the size of each experimental group is detailed in the figure legends.

### Locomotor Assessment (Basso-Bresnahan-Beattie Locomotor Scale)

Injured animals were evaluated in the open field using the Basso-Bresnahan-Beattie (BBB) locomotor scale, according to the indications published in the original articles [13], [14]. Briefly, animals were introduced daily to the open field and handled over 2 weeks before induction of SCI. Then, animals were exposed once per week to the open field to maintain them habituated to the test and two trained observers scored rats over four minutes on post-lesion days 7, 14, 30, 60 and 90. One person recorded all the data on a score sheet while the other kept the animal moving. Movements elicited by the touch of an examiner were not scored.

### Thermal Sensitivity Evaluation (Cold and Hot Plates)

The effect of cannabinoid antagonists treatment on thermal sensitivity was evaluated by hot and cold plate tests. Briefly, spinal cord injured rats were habituated to the test by placing them on the plate at room temperature during 5 minutes each week from the first week after injury. Until 60 days after injury rats could not perform the test as they did not place the paw plantar surface in the plate or they did not sense heat or cold stimuli and rats were on risk of being injured by long heat or cold exposures. During the tests, we measured the time of withdrawal of hind paw from the plate at a temperature of 48°C (hot plate) or 0°C (cold plate). We always performed hot plates 2 hours after evaluating their locomotor skills in the open field and cold plates on the following day. Each animal was tested twice, separated by a 30 minutes interval. Among 60 and 90 days after injury tests, rats were exposed weekly to the plates at room temperature during 5 minutes to maintain them habituated to the test.

### Magnetic Resonance Data Acquisition and Analysis

Rats were anaesthetized and transcardially perfused with 4% paraformaldehyde in 0.1 M phosphate buffer 90 days after injury. The spinal cord was dissected out and post-fixed for 4 hours in the same solution at 4°C. Spinal cords were placed in Fluorinert (Sigma, Madrid, Spain) and MRI was performed on a 4.7 T Bruker BioSpec system (Bruker, Karlsruhe, Germany) with a H^1^ surface coil. Three dimensional T2-weighted Fast Spin Echo images (T2W-3D) were acquired with the following parameters: repetition time (TR) 1650 ms, echo time (TE) 70 ms, number of averages (NA)  = 1, slice thickness 0.5 mm, 16 sagittal slices per sample, field of view (FOV) 2.56×1.28×0.8 cm^2^, and data matrix 256×128×16. T2W-3D hyperintense lesions in the spinal cord were identified and the volumes and lengths were measured with ImageJ software (National Institutes of Health, USA).

### Tissue Processing for Immunohistochemistry

After MRI studies spinal cords were immersed in a solution of 30% sucrose in 0.1 M phosphate buffer during 72 h at 4°C. Tissue blocks of 1 cm including the epicentre of the spinal cord lesion in the middle of the block were then embedded in OCT compound (Tissue-Tek, Sakura Finetek, Zoeterwoude, NL), frozen on dry ice and cut into 30 µm thick coronal sections on a cryostat, which were sequentially collected on slides. Tissue blocks of 1 cm including the lumbar enlargement were cut into 40 µm thick coronal sections on a vibratome. Sections were sequentially collected and stored in Olmos solution for further free-floating immunohistochemistry.

### Eriochrome Cyanine Staining

The slides were dried and warmed at 37°C, immersed in acetone for 5 minutes and stained in eriochrome cyanine solution for 30 minutes at room temperature (RT). Slides were rinsed in water, differentiated in 5% iron alum for 10 minutes at RT, rinsed again in water and fully differentiated in borax-ferricyanide solution for 10 minutes at RT. Slides were then dehydrated through graded ethanol solutions, immersed in xylene and coverslipped using DePeX mounting medium.

Serial sections taken every 1.2 mm from 3.6 mm rostral up to 3.6 mm caudal to the epicentre were used to quantify cross-sectional area, cyst area and white matter preservation. All measurements were performed with ImageJ software.

### Immunohistochemistry

Immunostaining was carried out as described previously [12] using primary antibodies against myelin basic protein (MBP 1∶1,000, Covance, Princeton, NJ, USA), Iba-1 (1∶300, Wako Pure Chemical Industries, Osaka, Japan), MHC-II (1∶200, Serotec, Oxford, UK) or serotonin (1∶5,000, Sigma, Madrid, Spain), and secondary Alexa Fluor-594 anti-rabbit IgG (1∶1000, Invitrogen, Barcelona**,** Spain), Alexa Fluor-488 anti-mouse IgG (1∶1000, Invitrogen). Control immunostaining in which the primary antibodies were omitted was performed in parallel. When performed, counterstainning was done with Hoescht 33,258 (1∶1000; Invitrogen).

### Optical Density Measurement of MBP

Optical density of MBP staining was measured as previously described [12]). Briefly, all slides were processed in parallel, and microphotographs were acquired in the same session using a Leica DMR microscope coupled to a Leica DC480 digital camera set to the same intensity and amplification of fluorescence. The levels of intensity were fixed to those at which the control sections without primary antibody gave no signal. Using NIH Image Analysis software (ImageJ), we transformed the pictures to grey scale and the mean grey values were measured in the preserved white matter surrounding the lesion.

### Quantification of Microglia/macrophages Density

All slides were processed in parallel for double immunohistochemistry against Iba-1 and MHC-II. Microphotographs were taken in a Leica SP5 confocal microscopy maintaining the same settings for laser beam intensity, photomultiplier gain and offset. Settings were established after checking that no signal is observed in control sections without primary antibodies and no crossover between fluorophores is detected. Acquired images were subjected to “color threshold” by ImageJ software and the resulting fraction area was measured in the preserved white matter around the epicentre. Threshold value was maintained through all the measurements and was empirically determined as that which best fitted to the morphology of the whole cell, including cellular bodies and processes.

### Quantification of Iba-1^+^/MHC-II^+^ Cell Number

Confocal images of slides processed for double immunohistochemistry against Iba-1 and MHC-II were acquired at 63x magnification and double positive cells were counted. At least 2 images per section were randomly taken and, whenever it was possible, up to 4 images per section were analyzed.

### Quantification of Serotonin Axonal Density

We performed free-floating immunohistochemistry against serotonin in 8 sections per animal corresponding to L1–L3 lumbar spinal levels. We acquired microphotographs of both right and left spinal ventral horns in a Leica SP5 confocal microscope under the same settings of laser beam intensity, photomultiplier gain and offset. Settings were established after confirming that there was no signal in control sections without primary antibody. Then, serotonin axonal density was quantified as described by Grider et al. (2006) [15]. Briefly, images were processed with *Feature J* pluggin for ImageJ to select the smallest Hessian values. The resulting images were transformed into binary images by automatic thresholding and occupied area was measured.

### Statistics

Graph Pad Prism 5.0 software was used to analyse data and to generate graphics. One-way ANOVA followed by Bonferroni post-test were applied to analyse the data showed in figure 1. Data presented in figure 2 were analyzed by non-parametric Kruskal-Wallis test followed by non-parametric Mann-Whitney test. Data in figures 3 and 4 were subjected to one-way ANOVA followed by Bonferroni post-test. Two-way ANOVA followed by Bonferroni post-test was applied to analyse data showed on figure 5. Data in figured 6, 7 and 8 were analyzed by one-way ANOVA followed by Bonferroni post-test.

## Results

### Spinal Cord Injury Induces a Rapid Increase of Endocannabinoids

We previously measured the accumulation of endocannabinoids in response to SCI at 1, 7 and 28 days after injury, and reported a rapid increase of AEA at 1 day and a delayed increase of 2-AG at 7 and 28 days after lesion [11]. Here, we have measured the levels of 2-AG and AEA in the first hours after SCI and detected a rapid and acute increase of 2-AG at 4 h after injury, while AEA levels rise up at 4 h and further augments at 14 h after injury (*Figure 1*). Thus, SCI induced a previously undetected accumulation of endocannabinoids at the early phase of secondary injury.

### Early Blockade of CB1 and/or CB2 Receptors Impairs Motor Function Recovery after Spinal Cord Injury

We administered at 20 minutes after spinal cord injury either a single injection of vehicle (n = 10), the CB1 antagonists/inverse agonists AM281 (n = 6), the CB2 antagonists/inverse agonists AM630 (n = 5) or the combination of AM281and AM630 (n = 10). We evaluated the locomotor skills of injured rats following the Basso-Beattie-Bresnahan open field scale (BBB) and we observed that cannabinoid antagonist-treated rats were unable to achieve the recovery observed in the vehicle-treated rats (*Figure 2*). No differences in the locomotor skills of vehicle- and antagonists-treated rats were observed at 7 and 14 days after injury, but at 30 days after injury, AM630- and AM281/AM630-treated rats BBB values were significantly lower. Vehicle-treated rats still improve at 60 days after injury, when their BBB performance reach a plateau, while antagonists-treated rats reached a plateau at 30 days after injury. Thus, BBB values of AM281-, AM630- and AM281/AM630-treated rats are significantly lower than those of vehicle-treated rats at 60 and 90 days after injury.

### Early Blockade of CB1 and/or CB2 Receptors does not Increase Thermal Hypersensitivity

While cannabinoid receptor agonists induce analgesia, antagonists do the opposite [16]. Although these effects are limited up to a few hours after agonist or antagonist administration, we evaluated whether early blockade of CB1 and/or CB2 receptors could result in long-term hypersensitivity conducting cold and hot plate tests. SCI results in cold and heat hypersensitivity (*Figure 3*) and early post-lesional treatment of rats with CB1 and CB2 antagonists did not produce notable changes: only a transient and small improvement in cold hypersensitivity was observed in AM281- and AM630-treated rats at 60 days after injury. Also we evaluated mechanical hypersensitivity/allodynia with dynamic von Frey filaments test and we did not observed any difference between vehicle- or antagonists-treated rats (data not shown).

### Early Blockade of CB1 and CB2 Receptor Enhances Secondary Damage

We determined the effect of early blockade of CB1 or/and CB2 receptors on the long-term outcome of the lesion by evaluating several indicators of pathology after SCI. We measured the extension of the lesion 90 days after injury by quantifying the oedema-related hyperintense signalling in T2W-3D MRI and observed that AM281/AM630 treatment increased volume and length of T2 hyperintense signals when compared with vehicle-treated rats (*Figure 4*). However, AM281 only increased lesion volume but not lesion length. Next, we performed eriochrome cyanine staining on coronal sections taken every 1.2 mm from the epicentre to 3.6 mm rostrally and 3.6 mm caudally and we quantified the crossectional area, cyst area and preserved white matter (*Figure 5*). Only CB1 antagonist AM281 increased the crossectional area of spinal cord in segments located 2.4 mm rostral and 3.6 mm caudal to the epicentre, but not in the epicentre itself (*Figure 5B*). However, only AM281/AM630-treated rats increased the cyst area, which is bigger at 2.4 mm caudally (*Figure 5C*), and reduced the preserved white matter, which is smaller at the epicentre and 1.2 mm caudally. Since it is generally accepted that the amount of white matter preservation at the epicentre is directly related to functional outcome after SCI [14], this could contribute to the worsening of motor outcome observed in AM281/AM630-treated rats.

### Early Blockade of CB1 and CB2 Receptors Increases Myelin Damage

We have previously shown that the endocannabinoid 2-AG administered 30 minutes after SCI reduces myelin loss [12]. In agreement with this, here we observe that the opposite treatment (CB1 and CB2 receptors antagonism by co-administering AM281 and AM630) increases myelin damage as measured by a decrease in MBP immunostaining in the epicentre at 90 days after lesion (*Figure 6*). Administration of only AM281 or AM630 did not affect MBP immunorreactivity.

### Early Blockade of CB1 and CB2 Receptors Results in Increased Chronic Microgliosis

Inflammation occurs immediately after SCI, evolves and persists chronically around the epicentre area [17]. One indicator of CNS inflammation is microglia, which after being activated proliferates and gets reactive; i.e., changes its morphology retracting its cellular processes and augmenting its body size [18]. We observed that only AM281/AM630-treated rats exhibited at 90 days after injury a significant increase in the number and reactivity of microglial cells around the epicentre when compared to vehicle-treated rats (*Figure 7A, B*). However, we observed an increase in the number of Iba-1^+^/MHC-II^+^ cells in the spinal cord of AM281-, AM630- or AM281/AM630-treated rats (*Figure 7C*). Noticeable, in AM281-treated rats we observed Iba-1^+^ cells exhibiting a high expression of MHC-II with a rounded morphology and devoided of processes, suggestive of blood-derived monocyte infiltratates. These cells were rarely observed in AM630- or AM281/AM630-treated rats. This may suggest that early blockade of CB1 or CB2 receptors after SCI results at long-term in subtle differences in spinal cord inflammation.

### Early Blockade of CB1 and CB2 Receptors Decreases the Number of Serotonergic Fibers in Lumbar Spinal Cord

Serotonin fibres in lumbar spinal cord mainly represent brain stem afferents involved in the modulation of locomotor circuitry [19]–[22]. We observed a decrease in the number of serotonin-positive fibres below the lesion level in the lumbar spinal cord of AM281-, AM630- and AM281/AM630-treated rats, although in the case of AM281-treated animals the changes failed to reach statistical significance (p = 0.06; *Figure 8*). Since there are virtually no serotonergic neurons in the cord, our result suggests that less serotonin descending fibres are able to cross the lesion to caudal segments of the spinal cord in the rats treated with CB1 and/or CB2 receptor antagonists.

## Discussion

Our results show that the rapid activation of the cannabinoid receptors by endogenous ligands after SCI is an endogenous protective response. We previously reported that the endocannabinods AEA and 2-AG are overproduced after SCI [11]. Specifically, we showed that AEA increases acutely at 1 day after SCI while its synthesizing enzyme mRNA, NAPE-PLD, augments and its degradative enzyme mRNA, FAAH, is reduced. This strongly suggests that AEA levels uprising was an active rather than a passive response. Similarly, 2-AG increases in a delayed fashion at 7 and 28 days after lesion while a strong upregulation of DAGLα mRNA (its synthesizing enzyme) is observed. In the present study we measured the levels of both endocannabinoids at earlier time points, from 30 minutes to 14 hours after injury, and we observed that AEA is already accumulated at 4 hours after injury, reaching at 14 hours the levels previously observed at 1 day after lesion. Although in the present work we have not measured the levels of NAPE-PLD and FAAH, it seems that we are observing the earlier stage of the active AEA production that we previously observed at 1 day after injury [11]. In addition, we also detected an increase of 2-AG at 4 h after lesion that comes back to sham levels at 14 h. Therefore, after SCI there are 2 peaks of 2-AG accumulation: the early acute increase showed in the present study and a sustained response in later sub-chronic/chronic periods of SCI [11]. We showed that 2-AG accumulation in the sub-chronic/chronic phase was an active response. However, we have no data that could unravel whether in the early acute phase occurs the same. In any case, we have observed a biphasic 2-AG accumulation in the spinal cord that could be modulating both early and late events that take place after SCI, while AEA seems to be restricted only to early processes occurring after SCI.

After incomplete SCI in rats, animals spontaneously recover some locomotor function [23]. Our results show that the early blockade of either CB1, CB2 or both receptors impairs this spontaneous locomotor recovery. In this regard, all the histopathological changes elicited by CB1 and/or CB2 receptors blockade have been previously related to locomotor recovery worsering. However, antagonizing CB1 or CB2 receptors produces less histopathological alterations that blocking both receptors at the same time. Thus, while the blockade of CB1 and CB2 receptors results at long term in i) a greater oedema expansion, ii) bigger cyst volume, iii) less white matter preservation, iv) worse myelin preservation and v) increased microgliosis, blocking CB2 receptors alone does not induce any of those histopathological alterations and CB1 blockade only produces a greater oedema expansion.

Conversely, other histopathological features that correlates with worse functional outcome after SCI are found after administration of either AM281, or AM630 or AM281/AM630. Thus, all antagonists-treated rats exhibit accumulation in the spinal cord of microglial/monocytic cells expressing MHC-II, which has been classically considered a marker of microglial activation and a marker of M1 neurotoxic macrophages [24]. This may be relevant, since immunomodulation is a potential therapeutic strategy for SCI [25]. Indeed, it has been reported that immunomodulation correlates with improvement of SCI rats after administration of a selective CB2 receptor ligand [26] and it has been demonstrated that immunomodulation is necessary for the locomotor improvement induced by cannabinoids in a murine model of Multiple Sclerosis [27].

Also, AM281, AM630 or AM281/AM630 administration reduces the number of serotonin fibres that innervate the lumbar spinal cord. In this regard, it has been shown that recovery of motor function after SCI is closely related with the availability of serotonin for the modulation of the locomotor circuitry in the lumbar spinal cord [22], [28]–[32].

Regarding which endocannabinoid could be eliciting the protection that is blocked by cannabinoid receptors antagonists, either 2-AG or AEA are agonists of CB1 and CB2 receptors and both of them have been reported to be neuroprotective [4], [6], [10], [12], [33], [34], although some studies have involved to AEA in neurodegeneration [35]. Anyhow, endocannabinoid production is modulated on demand after neuronal stimulation as a physiological counterbalance mechanism that is also activated in pathological conditions. Thus, endocannabinoid synthesis retrogradely regulates neuronal excitability and synaptic plasticity [36] as well as protects against excitotoxic damage induced by abnormal high spiking activity [4]. In traumatic brain injury, a condition related to SCI, 2-AG is also increased and its exogenous administration limits the extension of secondary damage [6]. In the same line of evidence, we have previously reported that a single early injection of 2-AG to spinal cord injured rats reduces the lesion size and protects from white matter damage [12].

Overall, our results show that very early after incomplete SCI occurs an increase of 2-AG and AEA that may activate CB1 and CB2 receptors as a protective and necessary response for a proper spontaneous recovery of locomotor function.
